# Identification and distribution of new candidate T6SS effectors encoded in *Salmonella* Pathogenicity Island 6

**DOI:** 10.3389/fmicb.2023.1252344

**Published:** 2023-08-17

**Authors:** Carlos J. Blondel, Fernando A. Amaya, Paloma Bustamante, Carlos A. Santiviago, David Pezoa

**Affiliations:** ^1^Facultad de Medicina y Facultad de Ciencias de la Vida, Instituto de Ciencias Biomédicas, Universidad Andrés Bello, Santiago, Chile; ^2^Laboratorio de Microbiología, Departamento de Bioquímica y Biología Molecular, Facultad de Ciencias Químicas y Farmacéuticas, Universidad de Chile, Santiago, Chile; ^3^Facultad de Medicina Veterinaria y Agronomía, Universidad de Las Américas, Santiago, Chile; ^4^Núcleo de Investigaciones Aplicadas en Ciencias Veterinarias y Agronómicas, Facultad de Medicina Veterinaria y Agronomía, Universidad de Las Américas, Santiago, Chile; ^5^Departamento de Ciencias Químicas y Biológicas, Universidad Bernardo O'Higgins, Santiago, Chile

**Keywords:** *Salmonella*, T6SS, SPI-6, effector, immunity protein

## Abstract

The type VI secretion system (T6SS) is a contact-dependent contractile multiprotein apparatus widely distributed in Gram-negative bacteria. These systems can deliver different effector proteins into target bacterial and/or eukaryotic cells, contributing to the environmental fitness and virulence of many bacterial pathogens. *Salmonella* harbors five different T6SSs encoded in different genomic islands. The T6SS encoded in *Salmonella* Pathogenicity Island 6 (SPI-6) contributes to *Salmonella* competition with the host microbiota and its interaction with infected host cells. Despite its relevance, information regarding the total number of effector proteins encoded within SPI-6 and its distribution among different *Salmonella enterica* serotypes is limited. In this work, we performed bioinformatic and comparative genomics analyses of the SPI-6 T6SS gene cluster to expand our knowledge regarding the T6SS effector repertoire and the global distribution of these effectors in *Salmonella*. The analysis of a curated dataset of 60 *Salmonella enterica* genomes from the Secret6 database revealed the presence of 23 new putative T6SS effector/immunity protein (E/I) modules. These effectors were concentrated in the variable regions 1 to 3 (VR1-3) of the SPI-6 T6SS gene cluster. VR1-2 were enriched in candidate effectors with predicted peptidoglycan hydrolase activity, while VR3 was enriched in candidate effectors of the Rhs family with C-terminal extensions with predicted DNase, RNase, deaminase, or ADP-ribosyltransferase activity. A global analysis of known and candidate effector proteins in *Salmonella enterica* genomes from the NCBI database revealed that T6SS effector proteins are differentially distributed among *Salmonella* serotypes. While some effectors are present in over 200 serotypes, others are found in less than a dozen. A hierarchical clustering analysis identified *Salmonella* serotypes with distinct profiles of T6SS effectors and candidate effectors, highlighting the diversity of T6SS effector repertoires in *Salmonella enterica*. The existence of different repertoires of effector proteins suggests that different effector protein combinations may have a differential impact on the environmental fitness and pathogenic potential of these strains.

## Introduction

The type VI secretion system (T6SS) is a multiprotein nanomachine composed of 13 structural components and various accessory proteins that deliver protein effectors into target cells through a contractile mechanism (Cherrak et al., 2019; Coulthurst, 2019). The T6SS needle, composed of an inner tube (made of a stack of Hcp hexamer rings) and comprising a trimer of VgrG and a PAAR protein, is wrapped into a contractile sheath formed by the polymerization of TssB/TssC subunits. These are assembled into an extended, metastable conformation (Silverman et al., 2013; Cherrak et al., 2019). Contraction of the sheath upon contact with a target cell or sensing cell envelope damage propels the needle toward the target cell (Brackmann et al., 2017). T6SS effector proteins are classified as either cargo or specialized effectors. Cargo effectors are delivered by non-covalent interaction with some core components (Coulthurst, 2019), while specialized effectors are additional domains of either VgrG, Hcp, or PAAR proteins (Durand et al., 2014; Whitney et al., 2014; Diniz and Coulthurst, 2015; Ma et al., 2017; Pissaridou et al., 2018).

The extensive repertoire of effector proteins makes the T6SS a highly versatile machine that can target prokaryotic or eukaryotic cells (Coulthurst, 2019; Monjarás Feria and Valvano, 2020). Among the antibacterial effector proteins, some target the peptidic or glycosidic bonds of the peptidoglycan (Ma and Mekalanos, 2010; Russell et al., 2012; Srikannathasan et al., 2013; Whitney et al., 2013; Berni et al., 2019; Wood et al., 2019), or the FtsZ cell division ring (Ting et al., 2018). These antibacterial effectors are encoded in bi-cistronic elements with immunity proteins (E/I pairs) that bind tightly and specifically to their cognate effector preventing self-intoxication and killing of sibling cells (Russell et al., 2012). Other T6SS effectors are eukaryote-specific, such as those targeting the actin or microtubule cytoskeleton networks (Monjarás Feria and Valvano, 2020), and others (known as trans-kingdom effectors) can target both bacterial and eukaryotic cells (Jiang et al., 2014). These effectors include those targeting conserved molecules (NAD^+^ and NADP^+^) and macromolecules (DNA, phospholipids) or forming pores in membranes (Whitney et al., 2015; Tang et al., 2018; Ahmad et al., 2019).

Many enteric pathogens (e.g., *Salmonella*, *Shigella*, and *Vibrio*) use the T6SS to colonize the intestinal tract of infected hosts (Sana et al., 2016; Chassaing and Cascales, 2018), while some strains of the gut commensal *Bacteroides fragilis* use their T6SSs only for competition against other Bacteroidales species (Coyne and Comstock, 2019). The T6SS is, therefore, a key player in bacterial warfare.

The *Salmonella* genus includes more than 2,600 serotypes distributed between species *S. enterica* and *S. bongori* (Issenhuth-Jeanjean et al., 2014), which differ in clinical signs and host range (Uzzau et al., 2000). Serotypes are defined based on variations in the somatic, flagellar and capsular antigens, according to the Kauffmann-White-Le Minor serotyping scheme (Grimont and Weill, 2007; Issenhuth-Jeanjean et al., 2014). Worldwide, *Salmonella* infections are responsible for 95.1 million cases of gastroenteritis per year (GBD 2017 Non-Typhoidal Salmonella Invasive Disease Collaborators, 2019). In addition, the World Health Organization (WHO) has also included *Salmonella* as a high-priority pathogen due to the emergence of strains with high levels of fluoroquinolone resistance (GBD 2017 Non-Typhoidal Salmonella Invasive Disease Collaborators, 2019). In *Salmonella*, 5 T6SS gene clusters have been identified within *Salmonella* Pathogenicity Islands (SPIs) SPI-6, SPI-19, SPI-20, SPI-21, and SPI-22 (Blondel et al., 2009; Fookes et al., 2011). These T6SSs are distributed in 4 different evolutionary lineages: T6SS_SPI-6_ belongs to subtype i3, T6SS_SPI-19_ to subtype i1, T6SS_SPI-22_ to subtype i4a, and both T6SS_SPI-20_ and T6SS_SPI-21_ belong to subtype i2 (Bao et al., 2019). Besides their distinct evolutionary origin, these five T6SS gene clusters are differentially distributed among distinct serotypes, subspecies, and species of *Salmonella* (Blondel et al., 2009).

**Figure 1:**
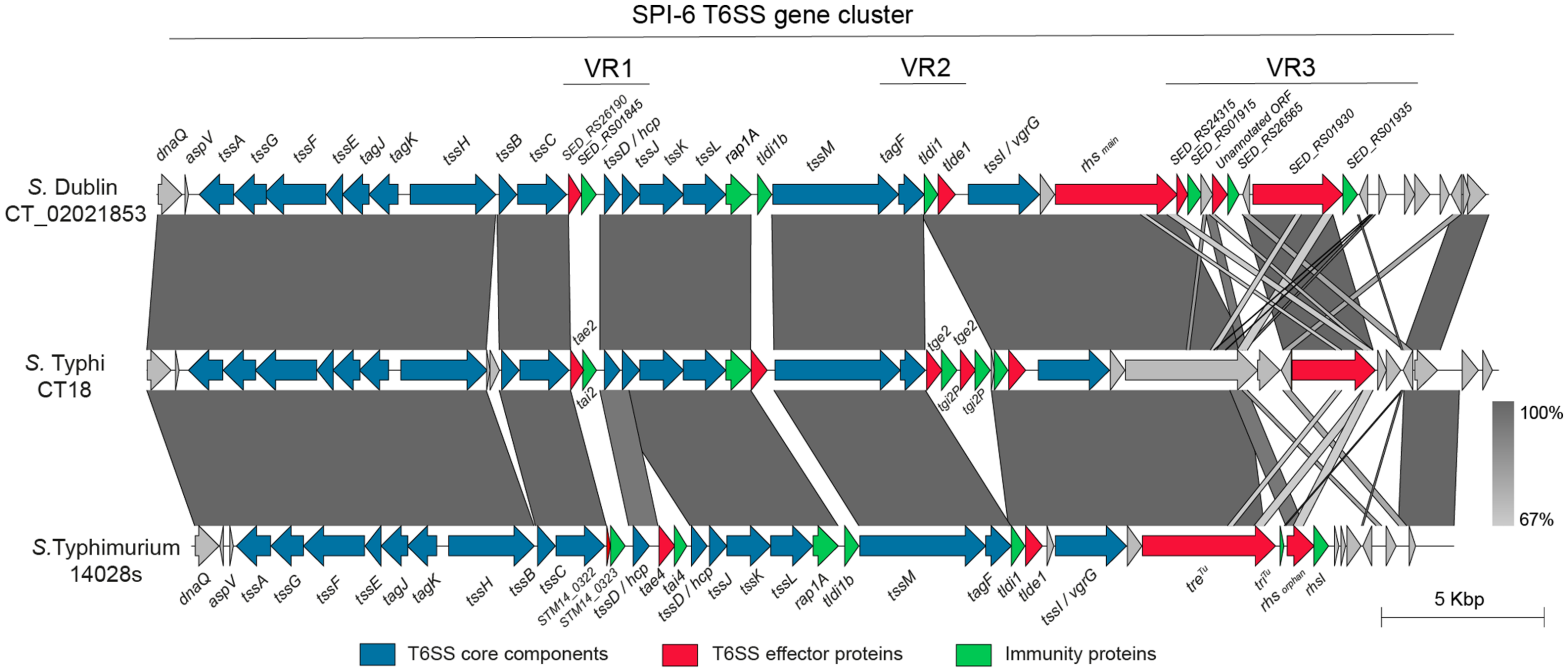
Schematic representation of selected SPI-6 T6SS gene clusters. The figure shows an alignment of the SPI-6 T6SS gene cluster of *S*. Dublin CT_02021853, *S.* Typhi CT18 and *S.* Typhimurium 14028s. The location of variable regions 1–3 is shown. ORFs encoding previously described T6SS effectors and cognate immunity proteins are shown in red and green, respectively. ORFs encoding T6SS core components are shown in blue. Grayscale represents the percentage of identity between nucleotide sequences.

Notably, most of these T6SSs have been shown to contribute to the virulence and pathogenesis of different *Salmonella* serotypes (Blondel et al., 2010; Mulder et al., 2012; Pezoa et al., 2013, 2014; Sana et al., 2016; Xian et al., 2020; Hespanhol et al., 2022; Sibinelli-Sousa et al., 2022). One of the most studied and widely distributed T6SS corresponds to that encoded in SPI-6. Depending on the serotype, the SPI-6 T6SS gene cluster comprises a region of ~35 to 50 kb encoding ~30 to 45 ORFs, including each of the 13 T6SS core components. The genetic architecture of the SPI-6 T6SS gene cluster is highly conserved among serotypes; nonetheless, there are structural differences restricted to three variable regions of the island (herein referred to as VR1, VR2, and VR3, Figure 1) (Blondel et al., 2009). In *S.* Typhimurium and *S*. Dublin, 9 SPI-6 T6SS effector proteins have been described to date (Russell et al., 2012; Benz et al., 2013; Whitney et al., 2013; Koskiniemi et al., 2014; Sana et al., 2016; Sibinelli-Sousa et al., 2020; Amaya et al., 2022; Jurėnas et al., 2022; Lorente-Cobo et al., 2022), most of which are encoded within these variable regions (Figure 1; Table 1).

**Table 1:** T6SS effectors and cognate immunity proteins encoded in SPI-6 previously identified in *Salmonella enterica.*

E/I pair	Effector activity	Paper highlights	References
**Effectors targeting peptidoglycan**			
Tae2/Tai2	Peptidoglycan hydrolase (Amidase that cleaves DD-crosslinks between D-mDAP and D-alanine)	Toxicity against the target-cell peptidoglycan in interbacterial competition	Russell et al. (2012)
Tae4-Tai4	Peptidoglycan hydrolase (Amidase that cleaves between D-mDAP and D-Glu)	Tae4 contributes to interbacterial competition and mice colonization	Sana et al. (2016)
Tge2/Tgi2P	Peptidoglycan glycoside hydrolase (N-acetylglucosaminidase)	Identified by bioinformatic analyses	Whitney et al. (2013)
Tlde1/Tldi1	Peptidoglycan L,D carboxypeptidase	Toxicity against the target-cell peptidoglycan in interbacterial competition	Sibinelli-Sousa et al. (2020)
**Effectors targeting nucleic acids**			
SED_RS01930/SED_RS01935	Ntox47 endonuclease (RNase)	SED_RS01930 contributes to interbacterial competition	Amaya et al. (2022)
SED_RS24315/SED_RS01915	Predicted Tox-URI2 endonuclease (DNase)	Identified by bioinformatic analyses	Amaya et al. (2022)
Unannotated ORF/SED_RS26565	Predicted HNH endonuclease (DNase)	Identified by bioinformatic analyses	Amaya et al. (2022)
Rhs^orphan^/RhsI	Ntox47 endonuclease (RNase)	Rhs^orphan^ contributes to bacterial killing during mice infection	Koskiniemi et al. (2014)
**Effectors targeting translation machinery**			
Tre^Tu^/Tri^Tu^	ART (ADP-ribosyltransferase)	Rhs^main^-type effector Tre^Tu^ arrests bacterial translation by ADP-ribosyltation of EF-Tu	Jurėnas et al. (2022)

The VR1 is located downstream of gene *tssC* and encodes the E/I modules Tae2/Tai2 and Tae4/Tai4. Tae2 and Tae4 are peptidoglycan hydrolases able to cleave the DD-crosslinks between D-mDAP and D-alanine or the covalent link between D-Glu and mDAP of the tetrapeptide stem, respectively, thus contributing to interbacterial competition and mice colonization (Russell et al., 2012; Sana et al., 2016). VR2 is located downstream of gene *tssM* and encodes many proteins of unknown function and two E/I modules with peptidoglycan hydrolase activity: Tge2/Tgi2P is predicted to have N-acetylglucosaminidase activity (Whitney et al., 2013), while Tlde1/Tldi shows L,D carboxypeptidase activity against the peptide stems of the peptidoglycan layer (Sibinelli-Sousa et al., 2020; Lorente-Cobo et al., 2022). Finally, the VR3 is located downstream of gene *tssI* and encodes a variable number of Rhs elements, some of them harboring endonuclease domains such as HNHc (DNase) and Ntox47 (RNase), and an ART domain (ADP-ribosyltransferase) linked to the C-terminal of these Rhs proteins (Koskiniemi et al., 2014; Amaya et al., 2022; Jurėnas et al., 2022).

Most of our knowledge regarding the presence and distribution of SPI-6 T6SS effector proteins comes from studies using reference strains of a limited number of serotypes (e.g., *S.* Typhimurium and *S.* Dublin) (Russell et al., 2012; Benz et al., 2013; Whitney et al., 2013; Koskiniemi et al., 2014; Sana et al., 2016; Sibinelli-Sousa et al., 2020; Amaya et al., 2022; Jurėnas et al., 2022; Lorente-Cobo et al., 2022). In this study, we performed a bioinformatic prediction analysis searching for putative T6SS effectors in a dataset of 60 genomes covering 37 *S. enterica* serotypes retrieved from the curated Secret6 database. Our analysis identified 23 new putative antibacterial effectors encoded in E/I modules within the 3 VRs of the SPI-6 T6SS gene cluster. These candidates include 5 effectors with putative peptidoglycan hydrolase activity, 16 effectors with potential nuclease activity and 2 effectors targeting the bacterial translation machinery. Finally, we expanded our analysis to include all available *Salmonella* genomes deposited in the NCBI database and determined the global distribution of these new putative effectors. A hierarchical clustering analysis identified that some effectors are conserved in most *Salmonella* serotypes. In contrast, most other effectors are differentially distributed in different serotypes. The presence of different sets of T6SS effectors suggests that distinct repertoires of these proteins may have a differential impact on the pathogenicity and environmental adaptation of *Salmonella* serotypes.

## Materials and methods

### Identification of candidate SPI-6 T6SS effectors

First, we searched the Secret6 database^1^ for *Salmonella* genomes encoding the minimal 13 core components of a T6SS and identified a total of 60 genomes that met this requirement. Then, to identify putative T6SS effectors encoded within SPI-6 of *Salmonella*, each ORF of this island was analyzed with the Bastion6 pipeline (Wang et al., 2018) excluding the 13 T6SS core components. ORFs presenting a Bastion6 score ≥ 0.7 were considered as candidate T6SS effectors. Each Bastion6 prediction was further analyzed with tools implemented in the Operon-Mapper web server (Taboada et al., 2018) to determine if it was likely part of a bi-cistronic unit also encoding a putative immunity protein [i.e., a small protein with potential signal peptides (SignalP 6.0) and/or transmembrane domains (TMHMM 2.0)]. Conserved functional domains and motifs in the candidate T6SS effectors were identified using the PROSITE, NCBI-CDD, Motif-finder, and Pfam databases (Kanehisa et al., 2002; Sigrist et al., 2013; Finn et al., 2014; Lu et al., 2019) implemented in the GenomeNet^2^ search engine. An e-value cutoff score of 0.01 was used. Finally, a biochemical functional prediction for each putative effector and immunity protein identified was performed by HMM homology searches using the HHpred HMM-HMM comparison tool (Zimmermann et al., 2017). It is worth mentioning that most genes (ORFs) identified do not have formal names, making extremely difficult referring to them using conventional genetic nomenclature. Thus, in figures and tables we will refer to ORFs encoding effectors and immunity proteins according to the corresponding protein name (in the case of those previously reported in the literature) or the functional domains present in the predicted proteins (in the case of ORFs encoding new candidate effectors and immunity proteins).

### Hierarchical clustering analysis of the new SPI-6 T6SS effectors

For hierarchical clustering analysis, a presence/absence matrix of each T6SS effector and candidate effector was constructed for each bacterial genome by means of BLASTn analyses and manual curation of the data. A 90% identity and 90% sequence coverage threshold was used to select positive matches. The matrix generated was uploaded as a csv file to the online server MORPHEUS^2^ using default parameters (i.e., one minus Pearson’s correlation, average linkage method).

### *Salmonella* 16S rDNA phylogenetic analyses

The 16S rDNA sequences were obtained from the 60 *Salmonella* genomes previously analyzed. The sequences were concatenated and aligned with ClustalW using the Molecular Evolutionary Genetics Analysis (MEGA) software version 7.0 (Kumar et al., 2016). A phylogenetic tree was built from the alignments obtained from MEGA by performing a bootstrap test of phylogeny (1,000 replications) using the maximum-likelihood method with a Jones-Taylor-Thornton correction model.

### Sequence and phylogenetic analyses

The DNA sequence encoding each T6SS effector identified in this study was subjected to BLASTn analyses to find orthologs in all *Salmonella* genome sequences deposited in the NCBI database (October 2022). For selection of positive matches, a 90% identity and 90% sequence coverage threshold was used. Conservation of sequences was determined by multiple sequence alignments using T-Coffee Expresso (Notredame et al., 2000), MAFFT (Katoh et al., 2017), and ESPript 3 (Robert and Gouet, 2014). Comparative genomic analysis of SPI-6 T6SS gene clusters was performed using Mauve (Darling et al., 2004) and EasyFig v2.2.5 (Sullivan et al., 2011). Nucleotide sequences were analyzed using Artemis version 18 (Rutherford et al., 2000).

## Results

### Analysis of a curated dataset of *Salmonella* genomes reveals 23 new putative E/I modules encoded within the SPI-6 T6SS gene cluster

To identify new T6SS effectors with high confidence, we first screened the SPI-6 T6SS gene clusters of a dataset of 60 *Salmonella enterica* genomes from the Secret6 curated database (Zhang et al., 2023). This database includes 60 strains covering 37 *Salmonella* serotypes (Supplementary Table S1). Each ORF within SPI-6 T6SS gene clusters was analyzed based on four criteria: (i) identification of candidate effectors through Bastion6 analysis (a bioinformatic tool that predicts T6SS effectors based on amino acid sequence, evolutionary information, and physicochemical properties); (ii) identification of putative immunity proteins by detection of signal peptides (SignalP 6.0), transmembrane domains (TMHMM 2.0) and operon prediction (Operon-mapper; Taboada et al., 2018); (iii) identification of conserved functional domains associated with *bona fide* T6SS effectors (INTERPROSCAN, PROSITE, NCBI-CDD, MOTIF, and Pfam) and (iv) functional biochemical prediction using the HHpred HMM-HMM server. In addition, we analyzed these gene clusters to identify potential unannotated ORFs which could encode putative effectors and cognate immunity proteins.

**Figure 2:**
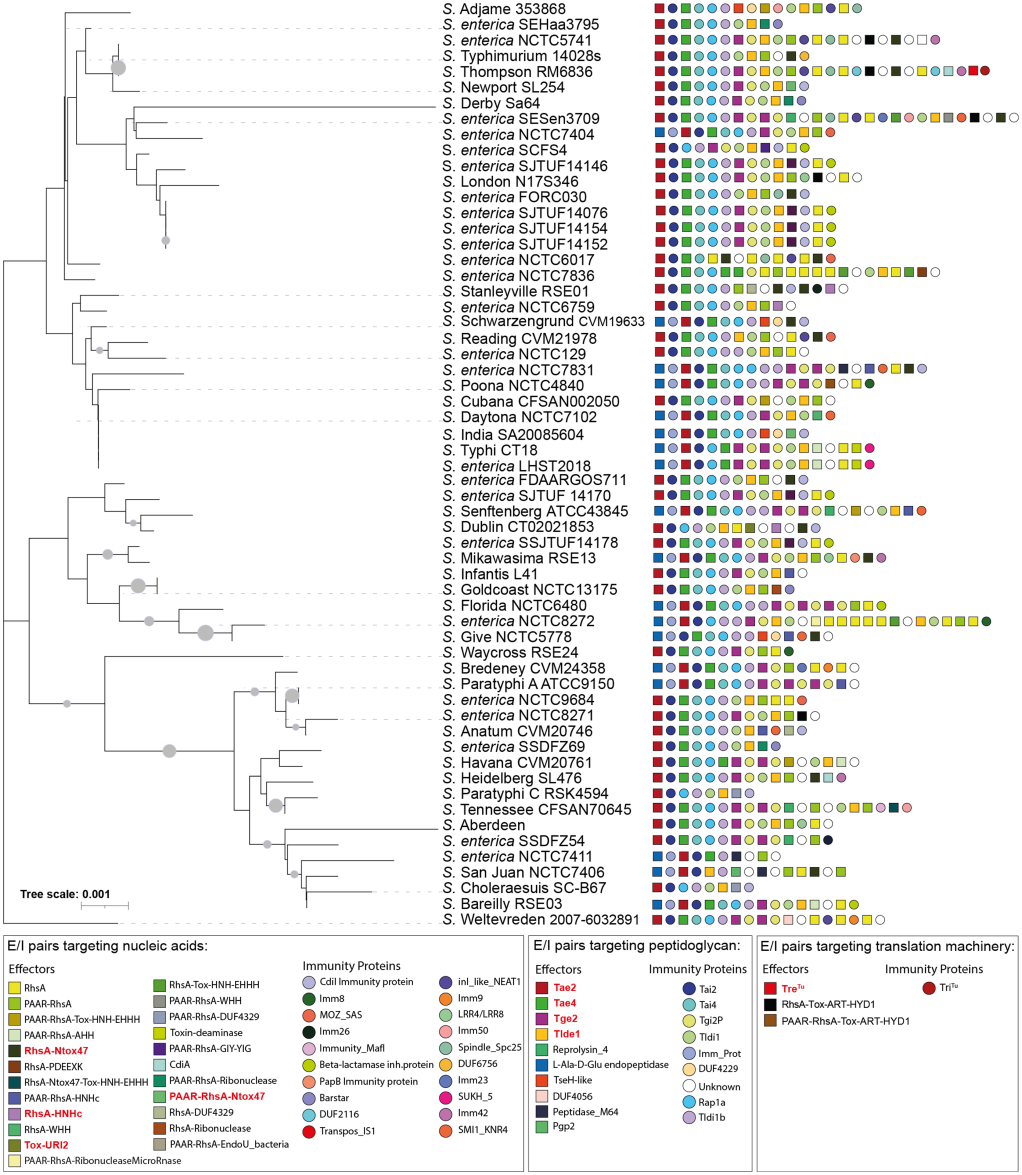
16S rDNA phylogeny and T6SS E/I module composition of *Salmonella enterica* SPI-6. Concatenated 16S rDNA nucleotide sequences from 60 *Salmonella* genomes deposited in Secret6 database were aligned with ClustalW using MEGA version 7.0. Next, a maximum-likelihood phylogenetic tree was built from the alignment using a bootstrap test of phylogeny (1,000 replications) with a Jones-Taylor-Thornton correction model. In the figure, we refer to ORFs encoding effectors and immunity proteins according to the corresponding protein name (in the case of those previously reported in the literature) or the functional domains present in the predicted proteins (in the case of ORFs encoding new candidate effectors and immunity proteins). Squares and circles next to each strain name correspond to ORFs encoding an effector or an immunity protein, respectively. Different colors represent confirmed or predicted functions, as indicated in the figure.

**Figure 3:**
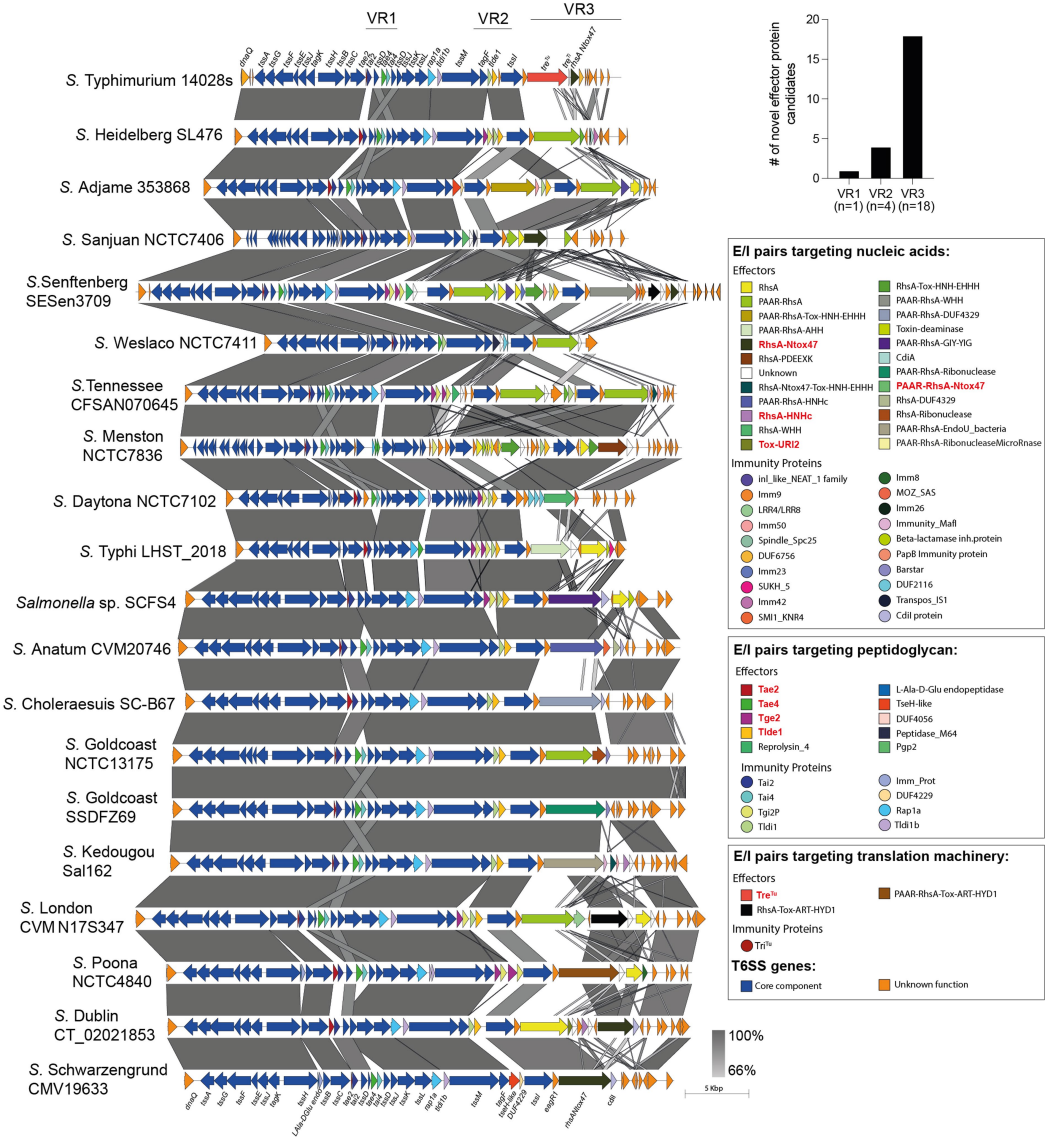
Comparative genomic analysis of SPI-6 T6SS gene clusters in representative *Salmonella* serotypes reveals new effector encoding genes. The location of variable regions 1–3 is shown. In the figure, we refer to ORFs encoding effectors and immunity proteins according to the corresponding protein name (in the case of those previously reported in the literature) or the functional domains present in the predicted proteins (in the case of ORFs encoding new candidate effectors and immunity proteins). ORFs encoding T6SS core components are shown in blue. ORFs encoding E/I modules are presented in different colors according to the confirmed or predicted functions, as indicated in the figure. Grayscale represents the percentage of identity between nucleotide sequences. Previously described *Salmonella* T6SS effectors are highlighted in red.

Our analysis identified 23 new putative effector proteins and cognate immunity proteins (Table 2). These candidates included both cargo and specialized effector proteins with diverse predicted biochemical functions, including peptidoglycan hydrolases (5), DNases (8), RNases (6), deaminases (1), ADP-ribosyltransferases (2) and hybrid DNases/RNases (1) (Table 2). In addition, our analysis showed that the repertoire of E/I modules in SPI-6 vary considerably between closely related strains (Figure 2). Of note, comparative genomic analyses revealed that each identified E/I module is encoded within one of the 3 VRs previously described (Blondel et al., 2009). One E/I module is encoded within VR1, four within VR2, and 18 are encoded within VR3 (Figure 3).

**Table 2:** New putative T6SS effectors and cognate immunity proteins encoded in SPI-6 of *Salmonella enterica.*

T6SS effector genes					Cognate T6SS immunity protein genes	
ORF(s)	Size (aa)	Serotype-strain	Variable region	Predicted activity/domain	ORF(s)	TM or signal peptide/domain
**Effectors targeting peptidoglycan**						
Unannotated ORF	32	*S*. Bareilly RSE03	1	Peptidoglycan hydrolase (Amidase)/L-Ala, D-Glu endopeptidase	ELZ70_17800	Signal peptide (Sec/SPI)/No
		*S.* Bredeney CVM24358			HFS03_00580	
		*S*. Daytona NCTC7102			NCTC7102_04795	
		*S.* Florida NCTC6480			NCTC6480_03851	
		*S*. Give NCTC5778			NCTC5778_03432	
		*S*. India SA20085604			Unannotated ORF	
		*S*. Mikawasima RSE13			Unannotated ORF	
		*S.* Paratyphi A ATCC9150			SPA_RS12640	
		*S*. Poona NCTC4840			NCTC4840_03690	
		*S. enterica* LHST_2018			Unannotated ORF	
		*S. enterica* NCTC7404			NCTC7404_03579	
		*S. enterica* NCTC7411			NCTC7411_03668	
		*S. enterica* NCTC7831			NCTC7831_03115	
		*S. enterica* NCTC8272			NCTC8272_03056	
		*S*. Sanjuan NCTC7406			NCTC7406_04092	
		*S*. Schwarzengrund CMV19633			SESA_RS02015	
		*S*. Senftenberg ATCC 43845			SEES3845_018760	
		*S.* Typhi CT18			STY_RS01380	
NCTC7406_04082	122	*S*. Sanjuan NCTC7406	2	Peptidoglycan hydrolase (L,D transpeptidase)/Pgp2	NCTC7406_04081	Signal peptide (Sec/SPI)/No
G9X22_18260	279	*S*. Adjame 353,868	2	Peptidoglycan hydrolase (Amidase)/TseH-like	Unannotated ORF	2 TM/DUF4229
NCTC5778_03416		*S*. Give NCTC5778			NCTC5778_03415	
LFZ16_04210		*S*. India SA20085604			LFZ16_04215	
SESA_RS02090		*S*. Schwarzengrund CMV19633			Unannotated ORF	
SESEF3709_03438	243	*S. enterica* SESen3709	2	Peptidoglycan hydrolase (Amidase)/Reprolysin_4	SESEF3709_03437	Signal peptide (Sec/SPI)/No
EOS97_RS15095		*Salmonella* sp. SSDFZ54			EOS97_RS15100	No/No
SEES3845_018655		*S*. Senftenberg ATCC 43845			SEES3845_018650	No/No
CS349_18880		*S*. Tennessee CFSAN070645			CS349_18875	No/No
NCTC7411_03656	243	*S. enterica* NCTC7411	2	Peptidoglycan hydrolase (Amidase)/Peptidase_M64	NCTC7411_03655	1 TM/No
NCTC7831_03138		*S. enterica* NCTC7831			NCTC7831_03139	1 TM/No
NCTC7406_04078		S. Sanjuan NCTC7406			NCTC7406_04077	1 TM/No
**Effectors targeting nucleic acids**						
Unannotated ORF	149	*S*. Kedougo Sal162	3	RNase and DNase/RhsA-Ntox47-Tox-HNH-EHHH	Unannotated ORF	No/Imm50
CS349_18795		*S*. Tennessee CFSAN070645			CS349_18790	
NCTC7836_04182	970	*S. enterica* NCTC7836	3	DNase/RhsA-PDEEXK	NCTC7836_04181	No/No
NCTC7836_04194	616	*S. enterica* NCTC7836	3	DNase/RhsA-Tox-HNH-EHHH	NCTC7836_04193	No/No
NCTC8272_03019	589	*S. enterica* NCTC8272			NCTC8272_03018	No/No
SESEF3709_03428	592	*S. enterica* SESen3709			SESEF3709_03427	No/Imm50
G9X22_18245	1,501	*S*. Adjame 353,868	3	DNase/PAAR-RhsA-Tox-HNH-EHHH	G9X22_18240	No/Imm50
CFSAN002050_RS06455	1,499	*S.* Cubana CFSAN002050			CFSAN002050_RS06460	No/No
HFQ57_17525		*S*. Havana CVM20761			HFQ57_17520	No/No
SEES3845_018630		*S*. Senftenberg ATCC 43845			Unannotated ORF	No/No
HFQ45_19990	1,377	*S*. Anatum CVM20746	3	DNase/PAAR-RhsA-HNHc	HFQ45_19995	No/SMI1_KNR4
NCTC5778_03412	1,382	*S*. Give NCTC5778			NCTC5778_03411	No/SMI1_KNR4
E4T58_01505	1,580	*S.* Infantis L41			E4T58_01510	No/No
SPA_RS12520	1,570	*S.* Paratyphi A ATCC9150			SPA_RS12515	No/No
NCTC7831_03143	1,382	*S. enterica* NCTC7831			NCTC7831_03144	No/SMI1_KNR4
SEES3845_018590	1,377	*S*. Senftenberg ATCC 43845			SEES3845_018585	No/SMI1_KNR4
NCTC7102_04762	1,044	*S*. Daytona NCTC7102	3	DNase/RhsA-WHH	NCTC7102_04761	No/SMI1_KNR4
SESEF3709_03422	1,575	*S. enterica* SESen3709	3	DNase/PAAR-RhsA-WHH	SESEF3709_03421	No/SMI1_KNR4
ELZ70_17690	1,354	*S*. Bareilly RSE03	3	DNase/PAAR-RhsA-AHH	ELZ70_17685	No/No
HFQ57_17490	1,368	*S*. Havana CVM20761			HFQ57_17485	
HI825_06260	1,317	*S. enterica* LHST_2018			HI825_06265	
STY_RS01485	1,354	*S.* Typhi CT18			STY_RS01490	
HF553_RS18710	1,374	*Salmonella* sp. SCFS4	3	DNase/PAAR-RhsA-GIY-YIG	HF553_RS18705	No/CdiI
HU143_RS17590		*Salmonella* sp. SJTUF14076			HU143_RS17585	
IVP14_RS17960		*Salmonella* sp. SJTUF14146			IVP14_RS17955	
IVP15_RS18860		*Salmonella* sp. SJTUF14152			IVP15_RS18855	
IVP16_RS17665		*Salmonella* sp. SJTUF14154			IVP16_RS17660	
IVP17_RS17925		*Salmonella* sp. SJTUF14170			IVP17_RS17920	
IVP18_RS17920		*Salmonella* sp. SJTUF14178			IVP18_RS17915	
HFQ45_20000	174	*S*. Anatum CVM20746	3	RNase/RhsA-DUF4329	HFQ45_20005	No/CdiI
ELZ68_18315	267	*S*. Stanleyville RSE01			ELZ68_18310	No/No
SCH_RS26875	1,593	*S.* Choleraesuis SC-B67	3	RNase/PAAR-RhsA-DUF4329	SCH_RS01475	No/CdiI
SPC_RS25995		*S.* Paratyphi C RSK4594			SPC_RS01475	
NCTC13175_03561	352	*S*. Goldcoast NCTC13175	3	RNase/RhsA-Ribonuclease/Microbial Rnase	NCTC13175_03560	No/Barstar
Unannotated ORF	1,564	*S*. Derby Sa64	3	RNase/PAAR-RhsA-Ribonuclease/Microbial Rnase	EIC79_17405	No/Barstar
SEBLO3795_03484	1,564	*S. enterica* SEHaa3795			SEBLO3795_03483	
EOS98_RS24920	1,560	*Salmonella* sp. SSDFZ69			EOS98_RS17735	
HLB37_13055	1,560	*S*. Kedougo Sal162	3	RNase/PAAR-RhsA-EndoU_bacteria	HLB37_13060	No/MafI
SEHA_RS26915	102	*S*. Heidelberg SL476	3	RNase/CdiA	SEHA_RS02130	No/Imm42
IA1_RS24740		*S*. Thompson RM6836			IA1_RS01635	
HI825_06280	106	*S. enterica* LHST_2018	3	Deaminase/Tox-Deaminase	HI825_06285	No/SUKH_5
STY_RS01505	86	*S.* Typhi CT18			STY_RS01510	
**Effectors targeting translation machinery**						
DYN42_004080	943	*S*. London CVM N17S347	3	ADP-ribosyltransferase/RhsA-Tox-ART-HYD1	DYN42_004085	No/No
NCTC8271_04564	194	*S. enterica* NCTC8271			Unannotated ORF	
NCTC5741_00975	943	*S. enterica* NCTC5741			NCTC5741_00976	
SESEF3709_03418	402	*S. enterica* SESen3709			SESEF3709_03417	
IA1_RS01605	959	*S*. Thompson RM6836			IA1_RS01610	
NCTC4840_03667	1,566	*S*. Poona NCTC4840	3	ADP-ribosyltransferase/PAAR-RhsA-Tox-ART-HYD1	NCTC4840_03666	No/No

### Putative T6SS cargo effectors with predicted peptidoglycan hydrolase activity are confined to VR1 and VR2

**Figure 4:**
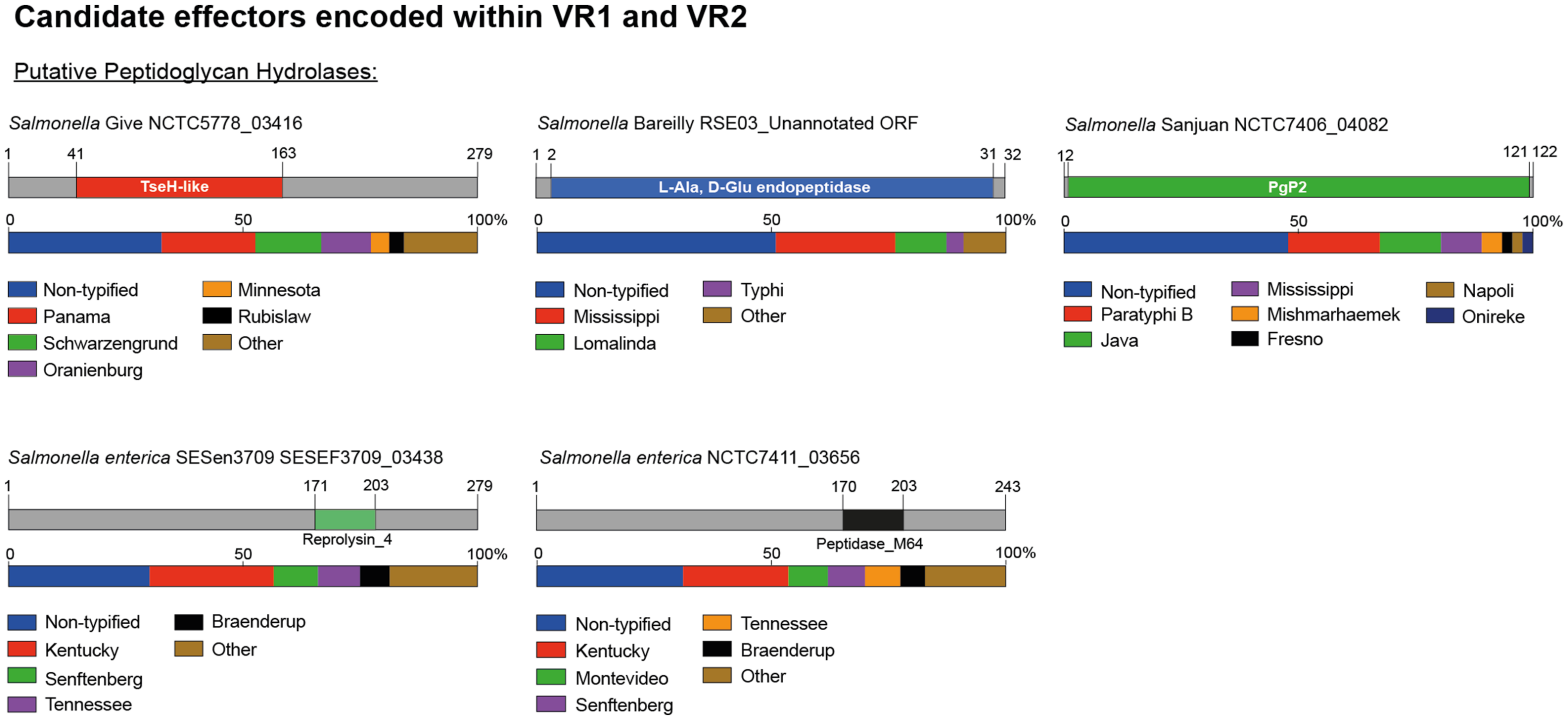
The variable regions 1 and 2 of the SPI-6 T6SS gene cluster encode 5 new putative effectors. Schematic representation and distribution of new putative effectors among *Salmonella* genomes. Predicted functional domains are show in different colors. Homologs for each candidate effector were identified by BLASTn analyses, as described in Materials and Methods.

Our bioinformatic analysis identified 5 predicted T6SS cargo effectors with putative peptidoglycan hydrolase activity (Table 2; Figure 4). One effector corresponds to an unannotated ORF encoded within VR1. This ORF was identified in 30% (18/60) of the genomes analyzed, is located between genes *tssH* and *tssB* (*ELZ70_17805* and *ELZ70_17795* ORFs in *S.* Bareilly strain RSE03) and is predicted to encode a 32 amino acids protein with a putative L-Ala-D-Glu-endopeptidase protein domain (Figure 4). This ORF is predicted to be co-transcribed with a downstream unannotated ORF that encodes a 146 amino acids protein with a periplasmic-targeting signal peptide (Table 2), suggesting that this latter ORF encodes the cognate immunity protein of the new candidate effector.

In addition, our analysis identified four putative E/I modules encoded in VR2. The first putative effector (NCTC7406_04082 in *S*. Sanjuan strain NCTC7406) is a 122 amino acid protein that harbors a predicted PgP2 protein domain with putative L,D transpeptidase activity (Table 2; Figure 4). *NCTC7406_04082* is part of a bi-cistronic unit with *NCTC7406_04081*. This latter ORF encodes a 147 amino acid protein with a signal peptide targeting the periplasmic space that may correspond to its cognate immunity protein (Table 2). The second VR2 candidate effector (G9X22_18260 in *S*. Adjame strain 353868) is a 279 amino acids protein that harbors a putative amidase domain similar to the NlpC/P60 endopeptidase domain of the TseH T6SS effector of *Vibrio cholerae* (Altindis et al., 2015). This candidate effector is also encoded next to a putative immunity protein of 86 amino acids harboring a DUF4229 protein domain and 2 transmembrane helices that may target this protein to the periplasmic space (Table 2).

The third candidate effector (SESEF3709_03438 in *S. enterica* strain SESen3709) is a 243 amino acids protein that harbors a Reprolysin_4 domain with putative amidase activity (Figure 4). *SESEF3709_03438* is predicted to be part of a bi-cistronic unit with *SESEF3709_03437*, that encodes a putative cognate immunity protein with a signal peptide for periplasmic targeting.

The final candidate effector of VR2 corresponds to a 243 amino acid protein with a predicted M64 peptidase domain (NCTC7411_03656 in *S. enterica* strain NCTC7411) (Table 2; Figure 4). Our analysis also revealed that *NCTC7411_03656* is likely to be part of bi-cistronic unit with their respective putative immunity protein gene (*NCTC7411_03655* in *S. enterica* strain NCTC7411) (Table 2). In other serotypes, the putative immunity protein gene encodes a protein of 84–144 amino acids harboring a transmembrane domain that targets this protein to the periplasmic space (Table 2).

### Putative T6SS specialized effectors with polymorphic nuclease and ADP-ribosyltransferase toxin domains associated to Rhs proteins are restricted to the VR3

**Figure 5:**
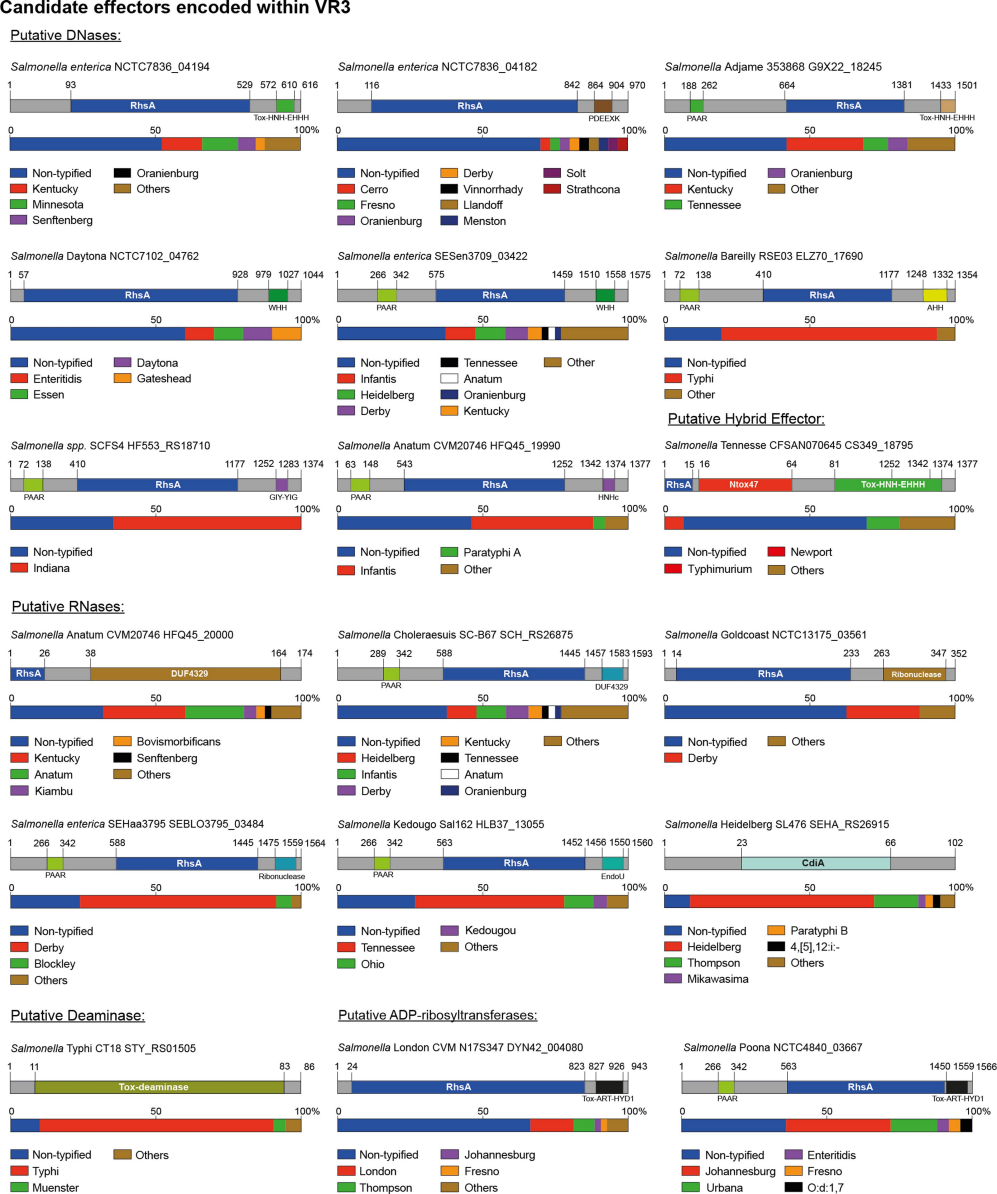
The variable region 3 of the SPI-6 T6SS gene cluster encodes 18 new putative effectors. Schematic representation and distribution of new putative effectors among *Salmonella* genomes. Predicted functional domains are shown in different colors. Homologs for each candidate effector were identified by BLASTn analyses, as described in Materials and Methods.

Our analysis revealed the presence of 18 candidate effectors encoded within the VR3 of SPI-6, including 16 in the Rhs family of proteins, 1 RNase and 1 deaminase. The size of the Rhs proteins ranged from 500 to 1,500 amino acids harboring different nuclease and ADP-ribosyltransferases domains (Table 2; Figure 5).

Eight of the 16 Rhs proteins harbored distinct C-terminal DNase domains, including domains of the HNH/ENDO VII superfamily of nucleases (IPR028048) such as WHH (IPR032869), Tox-HNH-EHH5H (IPR028048) or AHH (IPR032871), and nuclease domains of the GIY-YIG (IPR000305) and PDEEXK (IPR009362) families (Figure 5). In addition, 4 of these 8 candidates also harbored N-terminal PAAR motifs (IPR008727) (Figure 5). The presence of PAAR motifs suggests that these candidates correspond to specialized effector proteins. Each of these candidates were also predicted to be encoded in bi-cistronic units with ORFs encoding their respective immunity protein. Several of these proteins harbored domains previously found in cognate immunity proteins of bacterial toxin systems such as Imm50 (IPR028957), SMI1_KNR4 (PF09346) and CdI (IPR041256), among others (Table 2).

Our bioinformatics analyses also predicted 5 Rhs effectors with C-terminal extensions harboring different RNase protein domains (Table 2; Figure 5). These include Rhs proteins with Guanine-specific ribonuclease N1/T1/U2 (IPR000026), EndoU (IPR029501), and DUF4329 (IPR025479) domains. In addition, three of these proteins also harbored N-terminal PAAR motifs (IPR008727). The gene encoding each of these proteins was also predicted to be co-transcribed with genes encoding putative immunity proteins (Table 2). Remarkably, our analysis also identified a hybrid Rhs effector with predicted C-terminal RNase (Ntox47 domain) and DNase (Tox-HNH-EHHH) domains (CS349_18795 in *S*. Tennessee strain CFSAN070645). The gene encoding this protein is also predicted to be part of bi-cistronic unit with an ORF encoding a 129 amino acid protein with an Imm50 (IPR028957) domain. We also identified two putative Rhs effectors with a TOX-ART-HYD1 (pfam15633) ADP-ribosyltransferase domain, one of which also includes an N-terminal PAAR motif (NCTC4840_03667 in *S.* Poona strain NCTC4840). This protein shares 32% identity with STM0291, a recently described Rhs effector with an ART protein domain of *S.* Typhimurium named Tre^Tu^ (type VI ribosyltranferase effector targeting EF-Tu; Jurėnas et al., 2022). The low percentage of sequence identity (Supplementary Figure S1) suggests that this could be a divergent STM0291 homolog.

Finally, in VR3 we identified a putative effector with the CdiA RNase domain (IPR041620) not associated to Rhs elements (SEHA_RS26915 in *S*. Heidelberg SL476) (Table 2; Figure 5). In addition, we also identified a candidate effector harboring potential adenosine deaminase activity (STY_RS01505 in *S.* Typhi CT18). This effector is a small 86 amino acid protein with a TOX-deaminase domain of the BURPS668_1122 family (IPR032721) found in polymorphic toxin systems (Table 2; Figure 5). The gene encoding this effector is predicted to be co-transcribed with an ORF encoding a putative immunity protein with a SUKH_5 (PF14567) domain (Table 2; Figure 5).

### Genome-wide analysis of the distribution of SPI-6 T6SS effectors and candidate effectors in *Salmonella*

Identifying new putative T6SS effectors encoded within VR1-3 of SPI-6 encouraged us to determine the presence and distribution of the genes encoding these proteins across *Salmonella enterica*. The nucleotide sequence corresponding to each effector and candidate effector was used in BLASTn searches examining publicly available *Salmonella enterica* genome sequences deposited in the NCBI database, and the distribution of each effector protein was determined (Supplementary Table S2).

**Figure 6:**
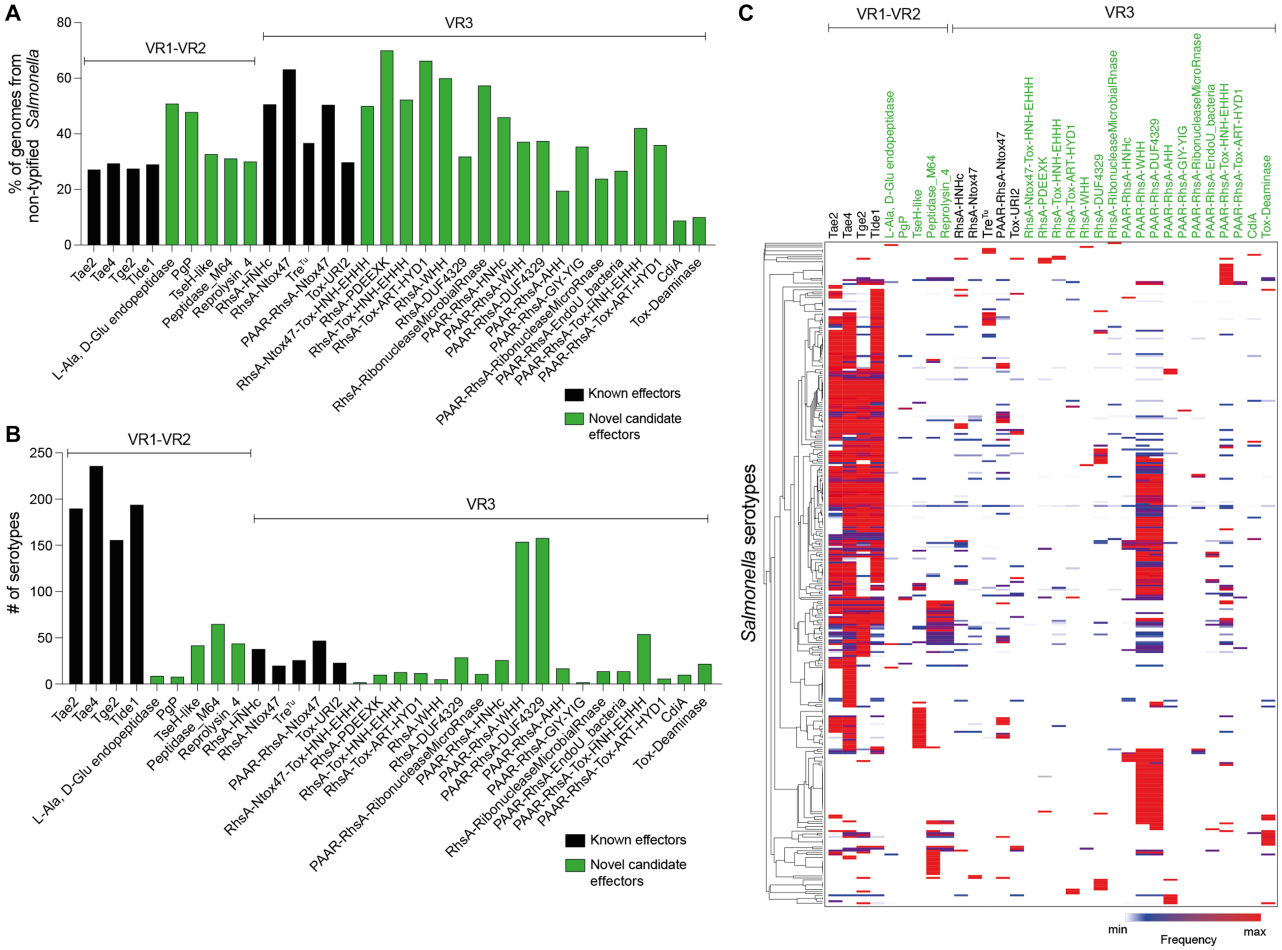
Prevalence of ORFs encoding T6SS effectors and candidate effectors in SPI-6. In the figure, we refer to ORFs encoding effectors according to the corresponding protein name (in the case of those previously reported in the literature) or the functional domains present in the predicted proteins (in the case of ORFs encoding new candidate effectors). Distribution of ORFs encoding T6SS effectors and candidate effectors in non-typified **(A)** and serotyped **(B)** *Salmonella* strains. **(C)** Prevalence of ORFs encoding T6SS effectors and candidate effectors in the genome of 340 *Salmonella* serotypes. A hierarchical clustering analysis was performed using MORPHEUS, as described in Materials and Methods. Color code in the heatmap indicates the presence of a given ORF (frequency) among all analyzed strains of a particular *Salmonella* serotype.

The analysis of the 9 T6SS effector proteins previously reported in the literature (i.e., Tae2, Tae4, Tge2, Tlde1, RhsA-HNHc, RhsA-Ntox47, PAAR-RhsA-Ntox47, Tre^Tu^ and Tox-URI2) and the 23 candidate effectors described in this study showed that they are widely and differentially distributed among *Salmonella* genomes (Supplementary Table S2). Interestingly, we identified these effectors and candidates effector in many non-typified *Salmonella* strains (Figure 6A).

Some effector and candidate effectors were more widespread across different serotypes than others (Figure 6B). Within VR1 and VR2, the previously reported effectors Tae2, Tae4, Tge2, and Tlde1 were identified across 150–240 serotypes, while the five candidate effector proteins identified in this study were found in 5–50 distinct serotypes. A different scenario was observed for effectors and candidate effectors encoded within VR3. In this case, the previously reported effectors were identified in less than 50 serotypes, while some new candidate effectors, such as PAAR-RhsA-WHH and PAAR-RhsA-DUF4329, were identified in over 150 serotypes. The distribution of each candidate effector in different *Salmonella* serotypes is highlighted in Figures 4, 5.

Finally, we performed a hierarchical clustering analysis to gain further insight into the distribution of effector and candidate effectors identified in 340 *Salmonella* genomes (Supplementary Table S3). As shown in Figure 6C, the four *bona fide* effectors encoded within VR1-2 (Tae2, Tae4, Tge2, and Tlde1) were the most conserved across the genomes of 113 different *Salmonella* serotypes. Nevertheless, these effectors are absent in the genome of 78 *Salmonella* serotypes, all of which include the genes encoding candidate effectors PAAR-RhsA-WHH and PAAR-RhsA-DUF4329 located within VR3. Furthermore, these candidate effectors are also distributed in the genome of 73 other *Salmonella* serotypes, suggesting that they play important roles in the biology of this pathogen.

## Discussion

The T6SS has emerged as an important virulence and environmental fitness factor for *Salmonella* (Blondel et al., 2010; Mulder et al., 2012; Pezoa et al., 2013, 2014; Sana et al., 2016; Xian et al., 2020; Hespanhol et al., 2022; Sibinelli-Sousa et al., 2022). However, information regarding the complexity and diversity of effector proteins for each distinct *Salmonella* T6SS is still lacking. In this context, even though the T6SS encoded in SPI-6 has been shown to contribute to host colonization by *S.* Typhimurium and *S*. Dublin (Mulder et al., 2012; Pezoa et al., 2013, 2014; Sana et al., 2016) and to interbacterial competition of *S.* Typhimurium against the intestinal microbiota (Sibinelli-Sousa et al., 2022), only 9 effector proteins have been identified and characterized so far (Russell et al., 2012; Benz et al., 2013; Whitney et al., 2013; Koskiniemi et al., 2014; Sana et al., 2016; Sibinelli-Sousa et al., 2020; Amaya et al., 2022; Jurėnas et al., 2022; Lorente-Cobo et al., 2022).

In this study, by means of bioinformatic and comparative genomic analyses, we identified a subset of 23 new SPI-6 T6SS candidate effectors, including peptidoglycan hydrolases, DNases, RNases, deaminases, and ADP-ribosyltransferases. Despite being well conserved, the SPI-6 T6SS gene cluster encodes a variable number of ORFs of unknown function restricted to three variable regions (VR1-3), that include the T6SS effectors previously identified in this species (Blondel et al., 2009). Notably, our analysis showed that every new T6SS effector identified is encoded within one of these variable regions. An interesting observation was that all predicted peptidoglycan targeting effectors are confined to VR1 and VR2. The reason behind this observation remains unclear; however, it is possible that VR1 and VR2 are hot-spots for gene recombination during *Salmonella* evolution, but the lack of mobile genetic elements surrounding these regions does not support this hypothesis. Importantly, in addition to the 4 peptidoglycan targeting effectors reported so far (Tae2, Tae4, Tge2, and Tlde1) (Russell et al., 2012; Benz et al., 2013; Whitney et al., 2013; Sana et al., 2016; Sibinelli-Sousa et al., 2020; Lorente-Cobo et al., 2022), we identified 5 candidate effectors encoded in VR1 and VR2 that presumably degrade peptidoglycan, indicating that this macromolecule is a common target site for *Salmonella* T6SS effectors. Of note, the unannotated ORF encoded in VR1 is the first putative effector that likely cleaves the link between L-Ala and D-Glu of the peptidoglycan peptide stems reported in *Salmonella* and shares homology to the peptidoglycan hydrolase ChiX of *Serratia marcescens* (30% identity and 45.2% similarity at amino acid sequence level) (Owen et al., 2018). This finding expands the peptidoglycan target sites exploited by *Salmonella* T6SS effectors against competing bacteria. On the other hand, the PgP2 and TseH-like candidate effectors are predicted to have redundant peptidoglycan degrading functions with other *Salmonella* T6SS effectors. PgP2 is predicted to have the same L,D transpeptidase exchange activity reported for Tlde1 (Sibinelli-Sousa et al., 2020; Lorente-Cobo et al., 2022), replacing D-Ala by a non-canonical D-amino acid preventing the normal crosslink between mDAP and D-Ala. In addition, the TseH-like candidate effector is a NlpC/P60 endopeptidase family protein (Xu et al., 2010; Altindis et al., 2015; Squeglia et al., 2019) predicted to cleave the covalent link between D-Glu and mDAP, as reported for Tae4 (Benz et al., 2013). These redundant functions suggests that the peptide stems are the main peptidoglycan target sites of *Salmonella* T6SS effectors, as only one identified effector targets the glycoside bonds in this macromolecule corresponds to Tge2 (Whitney et al., 2013). Remarkably, most serotypes encode combinations of T6SS effectors predicted to have hydrolytic activity toward different regions of the peptidoglycan structure. We hypothesize that this assortment of seemingly redundant effectors may improve the efficiency of the bacterial killing process.

The Reprolysin_4 domain found in some candidate effectors is present in zinc-binding metallo-peptidases harboring the binding motif HExxGHxxGxxH of family M12B peptidases. Of note, this motif is also present in the T6SS antibacterial effector SED_RS06335 with putative peptidoglycan hydrolase activity encoded in SPI-19 of *Salmonella* Dublin CT_02021853 (Amaya et al., 2022). The last candidate effector targeting the peptidoglycan identified in our study harbors the Peptidase_M64 protein domain that is also present in the IgA proteinase of *Clostridium ramosum* (Kosowska et al., 2002), recently reclassified as *Thomasclavelia ramosa* (Lawson et al., 2023). The putative immunity proteins of Reprolysin_4 and Peptidase_M64 have a signal peptide and a transmembrane domain, respectively. This suggests that both candidate effectors target the bacterial periplasm.

On the other hand, the VR3 of the SPI-6 T6SS gene cluster encodes a wide variety of effector proteins including domains found in DNases, RNases, deaminases and ADP-ribosyltransferases. Interestingly, most of these domains are fused to the C-terminal of Rhs proteins contributing to diversify the molecular targets of T6SSs in *Salmonella*. This was not unexpected since we have previously shown that the VR3 of SPI-6 encodes a variable number of Rhs elements (Blondel et al., 2009; Amaya et al., 2022) and many Rhs proteins have C-terminal polymorphic endonuclease domains associated with T6SS effectors in *Salmonella* and other bacteria (Zhang et al., 2012; Koskiniemi et al., 2014; Amaya et al., 2022). It is known that Rhs proteins have YD-peptide repeats, which fold into a large β-cage structure that surrounds and protects the C-terminal toxin domain increasing T6SS secretion efficiency (Donato et al., 2020; Jurėnas et al., 2021; Günther et al., 2022). This could explain why many T6SS effectors are associated to these elements.

Altogether, our work expands the repertoire of *Salmonella* T6SS effectors and provides evidence that the SPI-6 T6SS gene cluster harbors a great diversity of antibacterial effectors encoded in three variable regions. One interesting finding of our study is that peptidoglycan hydrolyzing effectors restricted to VR1 and VR2 are highly conserved in *Salmonella* genomes, while effectors targeting nucleic acids and the translation machinery encoded in VR3 are broadly distributed in *Salmonella* serotypes. This suggests that different repertoires of effectors could have an impact on the pathogenic potential and environmental fitness of these bacteria. Importantly, although this study increases the number of putative *Salmonella* antibacterial effectors against competing bacteria, we could not rule out that those targeting nucleic acids encoded in VR3 may also affect eukaryotic cells. This is an important knowledge gap, since no T6SS effector protein identified to date in *Salmonella* has been confirmed to target eukaryotic organisms, despite the clear contribution of *Salmonella* T6SSs to intracellular replication, survival and cytotoxicity inside the host immune cells (Mulder et al., 2012; Blondel et al., 2013; Schroll et al., 2019). Further research is required to address this issue. Finally, we are currently performing experimental work to confirm that each of the 23 candidates identified in our study correspond to *bona fide* T6SS effector proteins.

## Data availability statement

The original contributions presented in the study are included in the article/Supplementary material, further inquiries can be directed to the corresponding author.

## Author contributions

CB, FA, PB, CS, and DP: conceptualization, formal analysis, validation, writing-original draft preparation, writing review and editing, resources, project administration, and funding acquisition. CB and DP: methodology, investigation, and visualization. CS and DP: supervision. All authors contributed to the article and approved the submitted version.

## Funding

DP was supported by Fondo Concursable Proyectos de Investigación Regulares UDLA 2023 DI-13/23. CS was supported by FONDECYT grant 1212075. CB was supported by FONDECYT grant 1201805, ECOS-ANID ECOS200037 and HHMI-Gulbenkian International Research Scholar Grant #55008749. FA was supported by CONICYT/ANID fellowship 21191925.

## Conflict of interest

The authors declare that the research was conducted in the absence of any commercial or financial relationships that could be construed as a potential conflict of interest.

## Publisher’s note

All claims expressed in this article are solely those of the authors and do not necessarily represent those of their affiliated organizations, or those of the publisher, the editors and the reviewers. Any product that may be evaluated in this article, or claim that may be made by its manufacturer, is not guaranteed or endorsed by the publisher.

## Footnotes

^1^ https://bioinfo-mml.sjtu.edu.cn/SecReT6/download.html

^2^ https://www.genome.jp

^2^ https://software.broadinstitute.org/morpheus
