# Supplementary material for: Identification and distribution of new candidate T6SS effectors encoded in Salmonella Pathogenicity Island 6
Source: Front Microbiol. 2023 Aug 17;14:1252344. doi: 10.3389/fmicb.2023.1252344 (PMC10469887; doi:10.3389/fmicb.2023.1252344)
Supplement: Supplementary file 4 [file Data_Sheet_1.PDF]

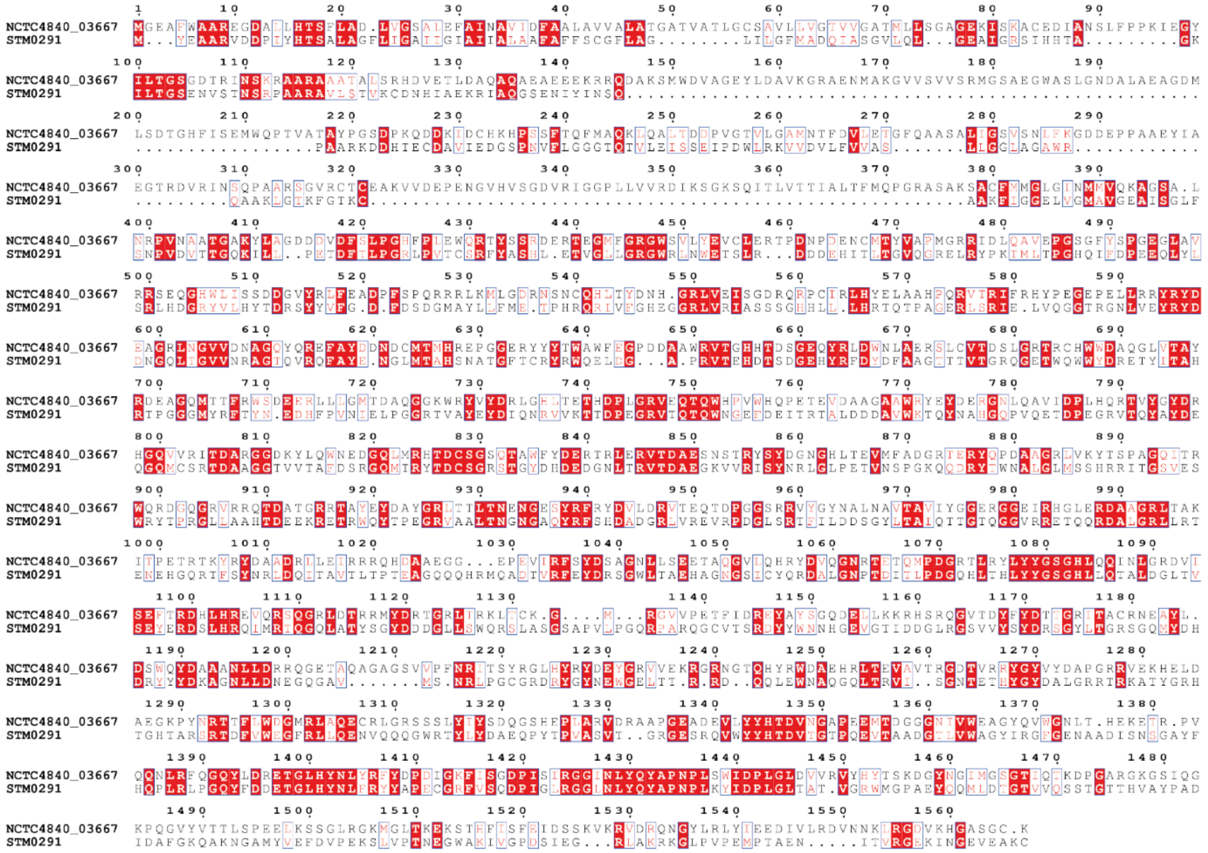

Score	Expect	Method	Identities	Positives	Gaps
531 bits(1367)	3e-167	Compositional matrix adjust.	407/1240(33%)	598/1240(48%)	91/1240(7%)

Figure S1. Sequence alignment of effector proteins NCTC4840_03667 from *S.* Poona NCTC4840 and STM0291 from *S.* Typhimurium LT2. A BLASTp alignment was performed using T-Coffee Expresso and visualized with ESPript 3. Identical amino acids are highlighted in boxes with a red background. Similar amino acids are highlighted in open boxes.
